# Sexual Dimorphic Distribution of Hypothalamic Tachykinin1 Cells and Their Innervations to GnRH Neurons in the Zebrafish

**DOI:** 10.3389/fendo.2020.534343

**Published:** 2021-03-03

**Authors:** Satoshi Ogawa, Priveena Nair Ramadasan, Rachel Anthonysamy, Ishwar S. Parhar

**Affiliations:** Brain Research Institute, Jeffrey Cheah School of Medicine and Health Sciences, Monash University Malaysia, Bandar Sunway, Malaysia

**Keywords:** Substance P, kisspeptin, gonadotropin-releasing hormone, preoptic area, reproduction

## Abstract

Substance P (SP) and neurokinin A (NKA), encoded by *TAC1*/*Tac1* gene are members of the tachykinin family, which exert their neuromodulatory roles in vertebrate reproduction. In mammals, SP and NKA have been shown to regulate gonadotropin-releasing hormone (GnRH) and luteinizing hormone (LH) secretion *via* kisspeptin neurons. On the other hand, the role of SP/NKA in the regulation of reproduction in non-mammalian vertebrates is not well known. In the present study, we first localized expression of *tac1* mRNA in the brain of male and female zebrafish, *Danio rerio*. Next, using an antibody against zebrafish tachykinin1 (Tac1), we examined the neural association of SP/NKA neural processes with GnRH3 neurons, and with kisspeptin (*kiss2*) neurons, in the brains of male and female zebrafish. *In situ* hybridization showed an apparent male-dominant *tac1* expression in the ventral telencephalic area, the anterior and posterior parts of the parvocellular preoptic nucleus, and the suprachiasmatic nucleus. On the other hand, there was female-dominant *tac1* expression in the ventral periventricular hypothalamus. Confocal images of double-labeled zebrafish Tac1 and GnRH3 showed associations between Tac1-immunoreactive processes and GnRH3 neurons in the ventral telencephalic area. In contrast, there was no apparent proximity of Tac1 processes to *kiss2* mRNA-expressing neurons in the hypothalamus. Lastly, to elucidate possible direct action of SP/NKA on GnRH3 or Kiss2 neurons, expression of SP/NKA receptor, *tacr1a* mRNA was examined in regions containing GnRH3 or Kiss2 neurons by *in situ* hybridization. Expression of *tacr1a* mRNA was seen in several brain regions including the olfactory bulb, preoptic area and hypothalamus, where GnRH3 and Kiss2 cells are present. These results suggest that unlike in mammals, Tac1 may be involved in male reproductive functions *via* direct action on GnRH3 neurons but independent of kisspeptin in the zebrafish.

## Introduction

Substance P (SP) and neurokinin A (NKA) are tachykinin peptides encoded by *TAC1*/*Tac1* gene, which are involved in a variety of biological actions in the central nervous system such as pain transmission (1), emotional behavior (2, 3), learning and memory (4, 5). In mammals, the *TAC1*/*Tac1* gene produces four different splicing variants (α-, β-, γ-, and δ-*Tac1*), which produce SP (encoded by α-, β-, γ-, and δ-*Tac1*), NKA (encoded by β- and γ-*Tac1*) and extended form of NKA peptide: neuropeptide K (NPK, encoded by β-*Tac1*) or neuropeptide gamma (NPγ, encoded by γ-*Tac1*) (6). Variant dependent expression of *Tac1* has been observed in different tissues, whereby β-*Tac1* and γ-*Tac1* mRNA are most abundantly expressed in the central nervous system (7). In the guinea-pig and rats, SP and NKA are co-expressed in the spinal ganglion cells and primary sensory neurons (8–10). Further, their co-release from the spinal cord has been shown in the guinea-pig (11). These results suggest that SP and NKA are synthesized together and function as co-transmitters in the same presynaptic terminals. SP and NKA preferentially bind to tachykinin receptors, NK1 receptor and NK2 receptor, respectively. In the brain, NK1 receptor is widely distributed, while NK2 receptor is sparsely but widely distributed in the peripheral nervous system (12).

In some mammals, SP and NKA have also been demonstrated as regulators of reproductive functions. In rodents, SP has been shown to stimulate gonadotropin-releasing hormone (GnRH) secretion and GnRH-induced luteinizing hormone (LH) release *via* NK1 receptor (13–16). Similarly, high doses of an agonist for NKA receptor (NK2 receptor) has been shown to stimulate GnRH pulse generation and LH release (17). However, in mice, NK1 receptor is expressed in 20% of GnRH neurons, and NK2 receptor is absent in GnRH neurons (16, 18), suggesting that SP/NKA may modulate GnRH-LH release *via* multiple mechanisms. GnRH neurons are known to be regulated by kisspeptin (Kiss1) and its receptor GPR54 (Kiss1 receptor) signaling, particularly during the onset of puberty. Recently, SP and NKA have been shown to modulate Kiss1 neurons and kisspeptin release (16, 19–21). In rodents, SP activates Kiss1 neurons (19) and approximately half of the Kiss1 neurons express NK1 receptor (16). Furthermore, *Tac1*-gene knockout male mice exhibit delayed puberty onset (22). In mammals, kisspeptin co-regulates GnRH neurons, together with other neuropeptides such as neurokinin B and dynorphin (KNDy hypothesis) (23). Therefore, SP/NKA could act as a potential regulator of the KNDy system to control the onset of puberty, although this may not be the case in primates and ewes (24, 25). Interestingly, it is well documented that SP neurons exhibit male-dominant expression pattern in the posterior-medial amygdala and basal ganglia regions (26–31). In addition, male rats have significantly greater concentrations of SP than females in preoptic-hypothalamic regions and the anterior pituitary (28, 32, 33). Male-dominant sexually dimorphic expression of SP has also been demonstrated in the diencephalic nucleus mainly in gymnotiform electric fish, *Apteronotus* sp. In *A. leptorhynchus*, SP-immunoreactive cell bodies in the lateral hypothalamus and their fiber innervations of the preoptic-hypothalamic areas are more abundant in the male, while no sex difference in SP-binding sites has been observed (34, 35). However, so far, the sexually dimorphic expression of SP/NKA has not been reported in other fish species, except for one report in goldfish showing sexually dimorphic (female dominant) expression of *tac1* mRNA in association with seasonal variations in the olfactory bulbar regions (36).

The role of SP/NKA in reproductive functions has also been demonstrated in non-mammalian vertebrates. In lizard, *Podarcis sicula*, SP has been shown to modulate brain aromatase activity, androgen and estrogen levels during reproductive behavior (37). In crested newt, *Triturus carnifex*, SP downregulates GnRH-induced LH secretion in cultured pituitary cells (38). In some teleosts, SP neurons directly innervate the pituitary (34, 39, 40). However, despite the evolutionarily conserved molecular structures of SP/NKA and their receptors, the role of SP/NKA signaling in reproductive functions has not been demonstrated in teleosts. In some teleosts, there are three GnRH (GnRH1, GnRH2, and GnRH3) (41, 42), while in others, there are two GnRH (GnRH2 and GnRH3) with multiple receptor types (43). In the zebrafish (*Danio rerio*), GnRH3 is the hypophysiotropic form since the ablation of GnRH3-expressing cells during early zebrafish development results in infertility (44). On the other hand, GnRH2 is involved in the regulation of food intake (45, 46). In addition, some teleosts possess multiple kisspeptin (Kiss1 and Kiss2) and their receptor types (43). In the brain of zebrafish, Kiss1 is predominantly expressed in the habenula which is involved in social functions, while Kiss2 present in the preoptic-hypothalamic regions regulates GnRH3-LH secretion (47, 48). In mammals, several morphological evidences show the central role of SP/NKA in the regulation of the gonadotropic axis. For example, SP immunoreactivity has been detected within Kiss1 neurons in the human infundibular nucleus (49). SP processes have been shown to make close apposition with kisspeptin (KNDy) neurons in the arcuate nucleus in rhesus monkey and goats (24, 50). In addition, Kiss1 and SP processes make occasional contacts with GnRH processes in the human postinfundibular eminence (51). However, neuronal and functional associations between SP/NKA and GnRH3 or Kiss2 remain unknown in non-mammalian vertebrates.

In the present study, we first examined the expression of *tac1* mRNA in the male and female zebrafish brain to confirm if there is any sexual dimorphism in *tac1* gene expression in the brain. Second, to visualize SP/NKA neural processes (dendrites, axons and axon terminals), we created an antibody against zebrafish prepro-tachykinin-1 (referred to here as Tac1). Using the zebrafish Tac1 antibody, we examined the association between SP/NKA neural processes and GnRH3 or Kiss2 neurons in the male and female brains of the zebrafish. Finally, we examined the expression of SP/NKA receptor (NK1 receptor, *tacr1a*) in the brain regions containing GnRH3 and Kiss2 neurons.

## Material and Methods

### Animals

Sexually mature (< 6 months) male and female zebrafish, *Danio rerio*, were maintained in freshwater aquaria at 27 ± 0.5°C under a controlled natural photo-regimen (14/10 h, light/dark). The fish were fed an Adult Zebrafish Diet (Zeigler, Gardners, PA, USA) twice daily. All experimental procedures were performed under the guidelines of the Animal Ethics Committee of Monash University (Approval number: MARP/2012/120).

### *In Situ* Hybridization of *tac1* mRNA

*In situ* hybridization was performed for the localization of *tac1* mRNA expressing cell population as described previously (52). Sexually mature male and female zebrafish (n = 4) were anesthetized by immersion in 0.01% solution of 3-aminobenzoic acid ethyl ester methanesulfonate (MS-222; Sigma, St. Louis, MO) before decapitation. The brains were fixed in 4% paraformaldehyde buffered in 0.2 M phosphate buffer (pH7.5) for 6 h, cryoprotected in 20% sucrose and embedded in Tissue Tek OCT compound (Sakura Finetechnical, Tokyo, Japan). Coronal sections of male and female zebrafish brain (n = 4) (15 µm thickness) were cut using a cryostat and were thaw-mounted onto 3-aminopropylsilane-coated glass slides. The sections were permeabilized with 0.2 M HCl for 10 min and were treated with proteinase K (1 μg/ml) for 15 min at room temperature, pre-hybridized at 55°C for 2 h, and hybridized with digoxigenin (DIG)-labeled 342 nucleotides (nt) fragments of zebrafish *tac1* riboprobe (50 ng/ml) covering 110-451 nt of zebrafish *tac1* mRNA [GenBank accession number: NM_001256391.1, retrieved from the National Center for Biotechnology Information (NCBI)] at 55°C overnight in a humidified chamber. The sections were then washed and blocked with 2% normal sheep serum. The DIG-labeled probe was detected using an alkaline phosphatase-conjugated anti-DIG antibody (Roche Diagnostics, Mannheim, Germany; 1:500 dilution), and chromogenic reaction was developed using 4-nitroblue tetrazolium chloride/5-bromo-4-chloro-3-indolyl-phosphate (Roche Diagnostics).

### Zebrafish Tac1 Antibody Creation

SP and NKA are encoded by the same gene, *tac1*, and they share a common carboxy-terminal amino acid sequence (Glu-Leu-Met) with other tachykinin family of peptides. Hence, some mammalian SP antibody may cross-react with other tachykinin peptides. Therefore, we created an antibody against preproTachyknin-1 (Tac1) to refer to SP and NKA peptides. An anti-zebrafish Tac1 polyclonal antiserum (Cat# PAS 15351/15352) was raised against a synthetic peptide (C-SYEWGTVQIYDKRRS) from the zebrafish *tac1* by 1st BASE Sdn Bhd (Selangor, Malaysia). The peptide was conjugated with keyhole limpet hemocyanin (KLH) as a carrier molecule and immunized in rabbits. Following the final injection, blood was obtained, centrifuged and the affinity-purified serum was stored at -80°C. The specificity of the immunoreactivity of the anti-zebrafish Tac1 antiserum was confirmed by i) immunohistochemistry using the antibody pre-absorbed with 10 µg/ml antigen peptide (1st BASE) or KLH and ii) dot-blot assay. The patterns of zebrafish Tac1 immunoreactive cells were also compared to those of zebrafish *tac1* mRNA expressing cells to validate the antibody specificity.

### Dot Blot Analysis for Tac1 Antiserum

Cross reactivities of the Tac1 antiserum, produced in our laboratory, was analyzed by dot blot analysis. Antigens, zebrafish Tac1 peptide (C-SYEWGTVQIYDKRRS) and mammalian (human) SP peptide (RPKPQQFFGLM, CAS 33507-63-0, Calbiochem) (10 µg) were spotted on a polyvinylidene difluoride (PVDF) membrane (Hybond-P, Amersham Biosciences, Buckinghamshire, England) and dried by air. The PVDF membranes were blocked with Tris-buffered saline (pH 7.4) containing 1% Tween-20 (TBST) buffer containing 0.5% nonfat dry milk for 2 h at room temperature, and incubated with Tac1 antiserum (1:1000), rabbit anti-mammalian SP antibody (#20064, ImmunoStar, 1:1000) and Tac1 antiserum preabsorbed with Tac1 antigen peptide (10 µg/ml in a final concentration) for overnight at 4°C. The membranes were washed with TBST and incubated with horseradish peroxidase-conjugated donkey anti-rabbit IgG (Amersham Biosciences) for 1 h at room temperature. Immunoreactivity was detected with ECL Plus Western Blotting Detection Reagents (Amersham Biosciences) and visualized with Light-Capture System attached with a cool CCD camera (ATTO, Tokyo, Japan).

### Immunohistochemistry for Tac1 in the Brain of Zebrafish

Immunohistochemistry was performed to examine the distribution of Tac1 in the brain of zebrafish. Coronal sections of male and female brains (n = 4) were pretreated with 0.3% hydrogen peroxide (H_2_O_2_) in methanol for 20 min followed by incubation in blocking solution containing 2% normal goat serum and 0.5% Triton X-100 in phosphate buffer saline (PBS) for 30 min at room temperature. The sections were then incubated with rabbit anti-zebrafish Tac1 antiserum (1:500 dilution) prepared in blocking solution overnight at 4°C in a moist chamber. The sections were incubated with biotinylated anti-rabbit IgG (1:200 dilution) (Vectastain ABC Elite kit, Vector Laboratories Cat# PK-6101) for 1 h followed by avidin-biotin peroxidase complex for 45 min at room temperature. Chromogenic development was achieved with 0.05% 3, 3’diaminobenzidine tetrahydrochloride with 0.03% H_2_O_2_ in 0.05 M Tris-HCl (pH 7.5). Upon development of staining, sections were dehydrated in a series of ethanol concentration followed by xylene and mounted with DPX mountant (Fisher Chemical, New Jersey, USA) and sealed with a coverslip.

### Intracranial Administration of Colchicine

To enhance Tac1-immunoreactivity in female brains, female fish were intracranially administered with colchicine according to a previously reported procedure (53, 54). Fish were anesthetized as described above and a pinhole was made in the skull covering the telencephalic region by using a needle tip, and colchicine (0.5μg/g body weight) or water (n=3 for each treatment) in a volume of 1 µl was administered through a 35-G needle. The fish was individually placed in a recovery tank, and the brain was collected 24h after administration, and processed for immunohistochemistry for Tac1 as described above.

### Double-Labeling for Tac1 Processes With GnRH3 and *kiss2* Cells

The double–immunofluorescence for Tac1 processes with GnRH3 was conducted in male and female zebrafish. Sagittal brain sections of male and female zebrafish (n = 3; 15 µm thickness) were subjected to double-immunofluorescence of Tac1 and GnRH3. Sections were incubated with rabbit anti-zebrafish Tac1 antiserum (1:500 dilution) prepared in blocking solution overnight at 4°C in a moist chamber followed by development with Alexa Fluor 594-labeled anti-rabbit IgG (dilution of 1:500, Life Technologies Cat# A11037). Sections were then washed with 0.01 M PBS, incubated with mouse monoclonal anti-GnRH antibody [#LRH-13; dilution 1:2000; a gift from Prof Wakabayashi, Gunma University, Japan] and developed with Alexa Fluor 488-labeled anti-mouse IgG (Invitrogen, 1:500 dilution). The specificity of LRH-13 antibody has been characterized (55), and it has been widely used to detect GnRH3 type in various teleosts including zebrafish (56–60).

To examine the association between Tac1 processes and *kiss2* cell soma, double-labeling combining immunofluorescence (Tac1) and fluorescence *in situ* hybridization (*kiss2*) was carried out in male and female brains (n=3). Briefly, coronal brain sections were subjected to DIG-*in situ* hybridization for *kiss2* (47) as described above. Following hybridization, the sections were incubated with horseradish peroxidase-conjugated anti-DIG antibody (Roche Diagnostics, Cat# 11207733910) diluted 1:500 in Tris-NaCl-Tween (TNT) buffer consisting of 0.1M Tris-HCl (pH7.4), 0.15M NaCl and Triton X-100 with 1% normal goat serum for 2 h at room temperature. The DIG-labeled antibody was detected using Tyramide Signal Amplification (TSA) Plus Fluorescein kit (PerkinElmer/NEN Life Science Products, Wellesley, MA) by adding 1:50 dilution of reconstituted fluorescent tyramide to Plus amplification diluent (PerkinElmer/NEN Life Science Products). After the fluorescence *in situ* hybridization for *kiss2* mRNA, immunofluorescence labeling for Tac1 was performed as described above. Sections were then mounted with VECTASHIELD mounting medium (Vector Laboratories, Burlingame, CA).

### *tacr1a* mRNA Expression in the Brain Regions Containing GnRH3 and *kiss2* Cells

To elucidate the possible expression of SP/NKA (NK1) receptor in GnRH3 neurons, expression of NK1 receptor (*tacr1a-202*) mRNA was examined in the brain regions containing GnRH3 (forebrain) or *kiss2* neurons (hypothalamus) by DIG-*in situ* hybridization. *In situ* hybridization on sagittal (for GnRH3) or coronal (for *kiss2*) brain sections (n=3, males) were conducted using zebrafish *tacr1a* mRNA probes (520 nt, positioned 624-1144 nt, GenBank accession numbers: NM_001270478) as described above. Presence of GnRH3 and *kiss2* cells were confirmed in the respective alternate consecutive serial sections by immunohistochemistry or *in situ* hybridization as described above.

### Image Acquisition

For Tac1-immunostaining and *tac1*-*in situ* hybridization, the stained brain sections were scanned on a MIRAX MIDI Digital Slide scanner (Carl Zeiss, GmbH, Gottingen, Germany), and images were captured using the MIRAX Viewer Image Software (3DTech, Budapest, Hungary) at a 230 nm resolution with a 20× objective. Double-labeled fluorescence images were captured and analyzed using a confocal laser scanning microscope (C1si, Nikon Instruments) and two separate images were superimposed using computer software (NIS Elements BR, Nikon Instruments). The red channel was then converted to magenta, and brightness and contrast adjustments were made in Adobe Photoshop CC (Adobe, San Jose, CA, USA). Nomenclature for the zebrafish brain regions was adopted from (61–66).

### Cell Counting and Statistical Analysis

For manual counting of DIG-labeled *tac1* expressing cell numbers (n = 4 for males and females), an average of 4–5 consecutive sections/region for each sample were used. Numbers of *tac1* cells were counted in regions showing apparent sexually dimorphic expression of *tac1* including the anterior and posterior parts of the parvocellular preoptic nucleus, suprachiasmatic nucleus, ventral zone of the periventricular hypothalamus, and superior raphe. *tac1* cells were also counted in regions showing no sexually dimorphic expression, which included the periventricular nucleus of the posterior tuberculum, dorsal zone of the periventricular hypothalamus, midbrain region, and the periventricular gray zone of the optic tectum.

For manual counting of GnRH3 cells numbers, associated with or without Tac1 processes (n = 3 for males and females), an average of 10 sections/region [the ventral nucleus of ventral telencephalic area (Vv) and olfactory-terminal nerve (OB-TN)] for each sample were used. The percentage of GnRH3-immunoreactive cells accompanied by Tac1 processes was calculated.

Close associations between Tac1 processes and GnRH3 cell soma were defined according to the procedure previously reported (67). Briefly, the confocal images were captured with a 60× water immersion objective (numerical aperture = 1.2) with an additional 9.9× optical zoom to give a final magnification of 594×, which yielded a voxel size of 0.08 μm. The close association was quantified by calculating the limit of the image by optical resolution using the wavelength formula λ/2 × (numerical aperture) and finally determining the pixel size of each cube that existed between the two labeled processes to confirm whether there was any blank pixel present between them (68).

A single-blind study was used to count the cell numbers. No sample calculation was performed to predetermine the sample size. All values are presented as means (± SEM). The mean difference of cell numbers or percentage of cells between males and females were analyzed by unpaired Student’s *t*-test and size effects are reported as Cohen’s *d* with the significance set at P < 0.05.

## Results

### Distribution of *tac1* mRNA and Tac1 Immunoreactivity in the Male and Female Brain

**Figure 1:**
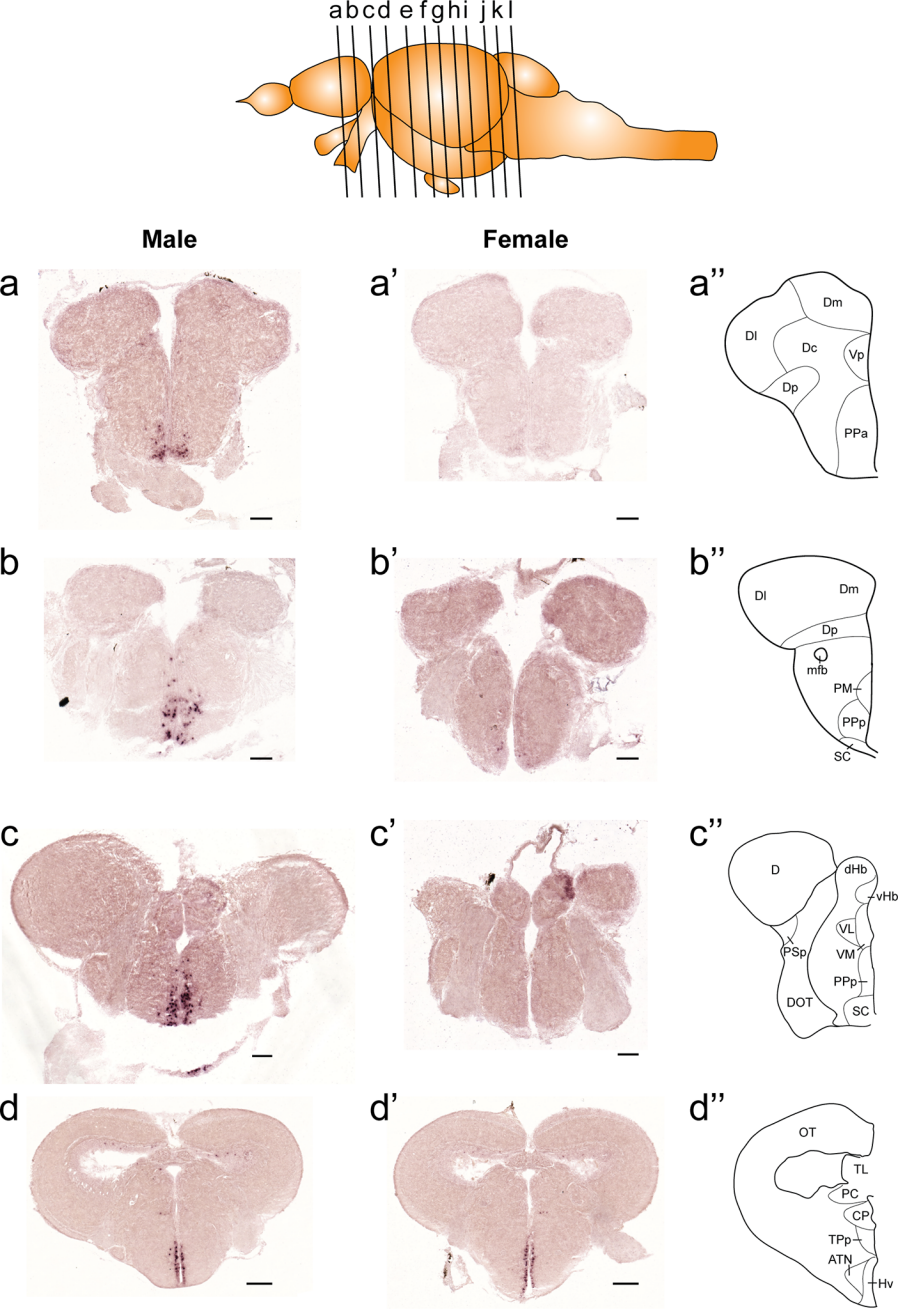
Comparison of expression patterns of *tac1* mRNA in the forebrain regions of male **(A−D)** and female **(A’−D’)** zebrafish. In the top panel, a schematic drawing of an adult zebrafish brain (lateral view) showing levels of cross-sections presenting in **Figures 1−3**. Alphabets represent respective figure numbers (**A−D**: **Figure 1, E−H: Figure 2**, and **I−L: Figure 3**). In the most-right panels **(A”−D”)**, a schematic drawing of half of the brain section (left side) processed for *tac1* staining indicating major brain structures at this level. There was a sexually dimorphic expression of *tac1* in the anterior part of the parvocellular preoptic nucleus **(A**, **A’)**, posterior part of the parvocellular preoptic nucleus **(B**, **B’)**, and suprachiasmatic nucleus **(C**, **C’)**. In contrast, no sexually dimorphic expression of *tac1* was observed in the dorsal habenula **(C**, **C’)** and the ventral region of the periventricular hypothalamus **(D**, **D’)**. Scale bars: **(A**–**C)**, **(A’**–**C’)**, 100 µm; **(D**, **D’)**, 200 µm. See Glossary for abbreviation.

**Figure 2:**
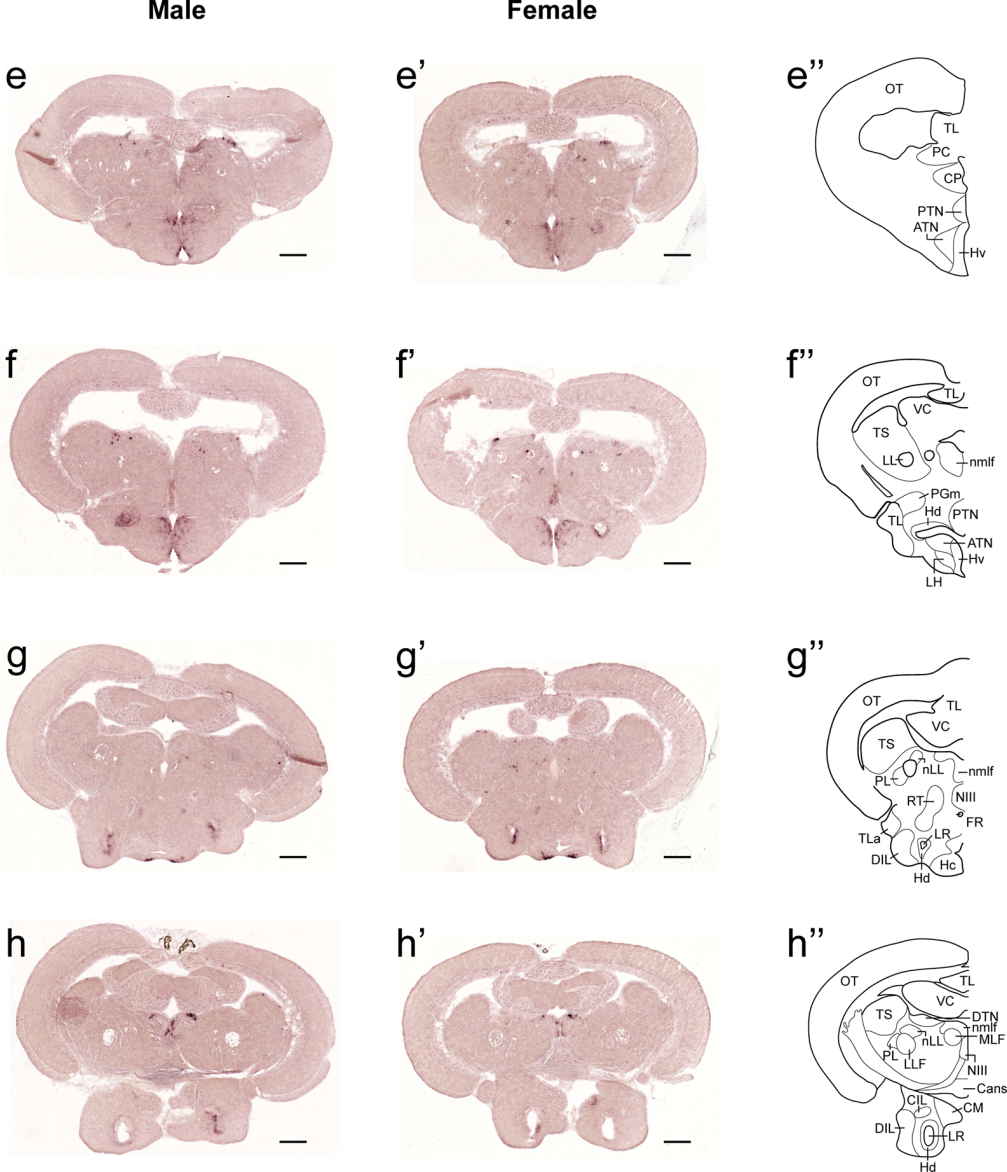
Comparison of expression patterns of *tac1* mRNA in the midbrain regions of male **(E−H)** and female **(E’−H’)** zebrafish. In the most-right panels **(E”−H”)**, a schematic drawing of half of the brain section (left side) processed for *tac1* staining indicating major brain structures at this level. There was no sex difference in expression of *tac1* in the diencephalic and mesencephalic regions including the torus semicircularis, dorsal tegmental nucleus, posterior tuberal nucleus, caudal and dorsal zones of the periventricular hypothalamus, nucleus of medial longitudinal fascicle, and oculomotor nucleus. Scale bars: 200 µm. See Glossary for abbreviation.

**Figure 3:**
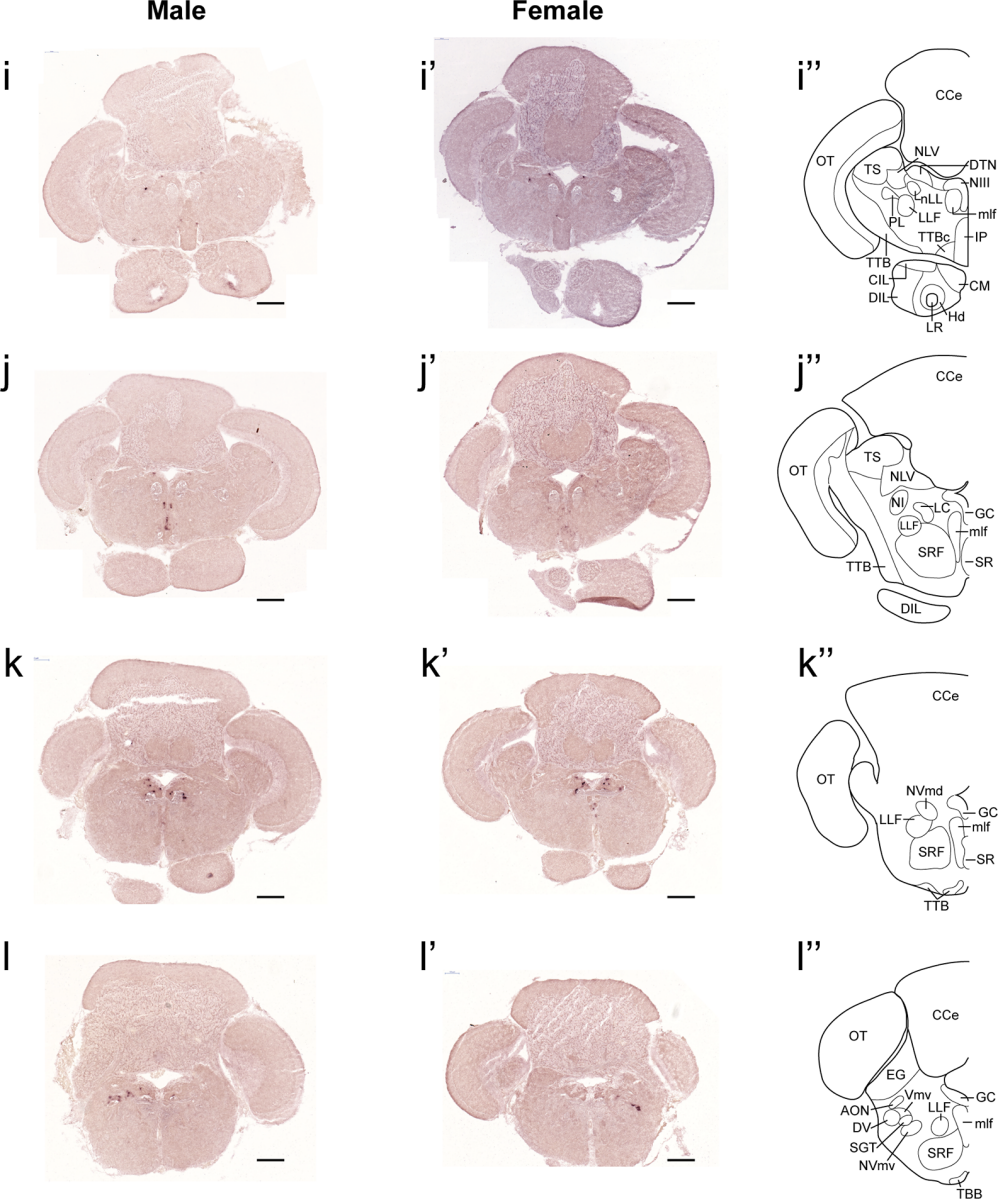
Comparison of expression patterns of *tac1* mRNA in the hindbrain regions of male **(I−L)** and female **(I’−L’)** zebrafish. In the most-right panels **(I”−L”)**, a schematic drawing of half of the brain section (left side) processed for *tac1* staining indicating major brain structures at this level. There was no sex difference in expression of *tac1* in the mesencephalic and rhombencephalic regions including the interpeduncular nucleus, dorsal tegmental nucleus, central gray, and trigeminal motor nucleus, while in the superior raphe, there was more *tac1* expression in males as compared to females **(J**, **J’)**. Scale bars: 200 µm. See glossary for abbreviation.

**Figure 4:**
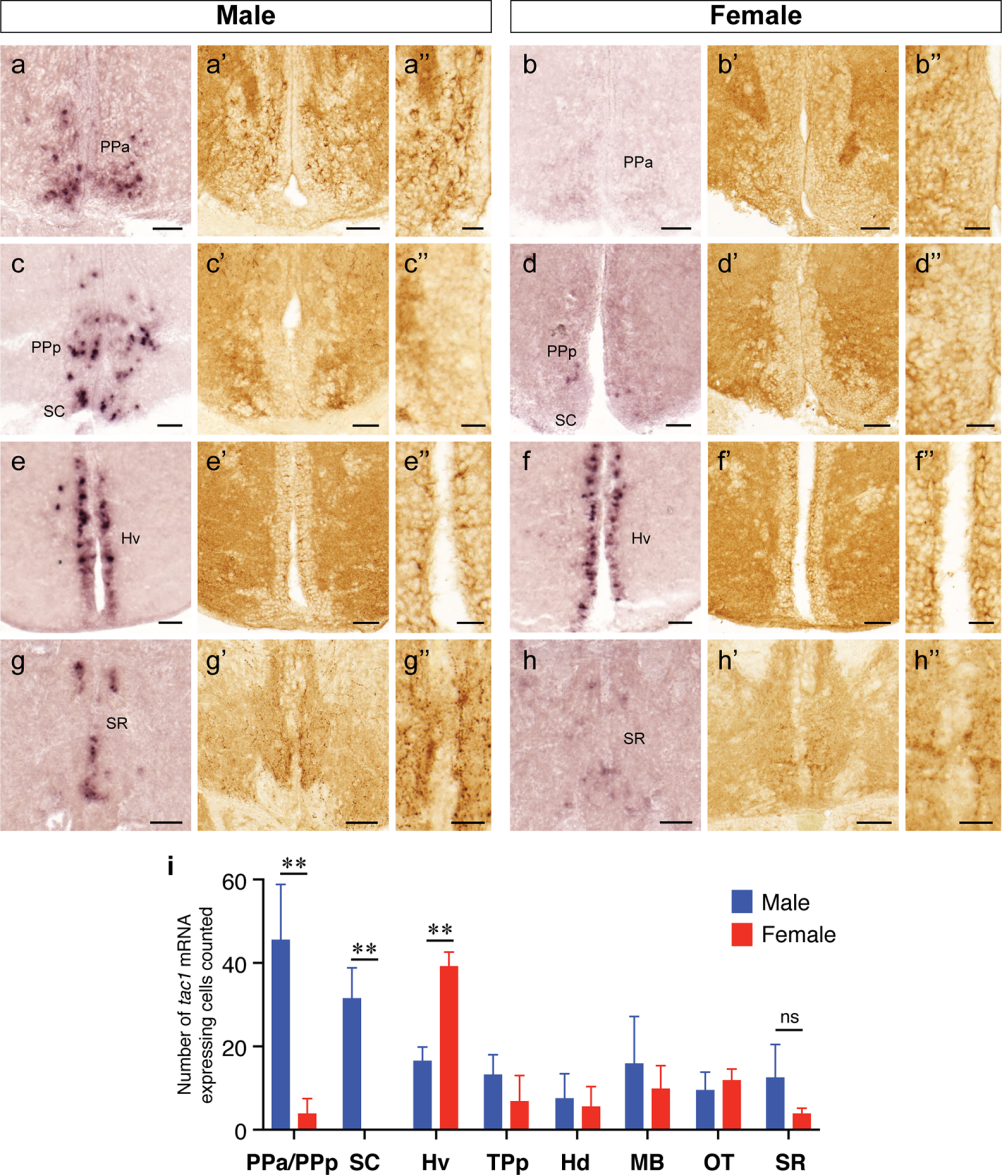
Sexually dimorphic expression of *tac1* mRNA and Tac1 immunoreactivity in the brain. Photomicrographs in the left **(A−H)**, middle **(A’−H’)** and the right **(A”−H”)** columns for each gender are *tac1* mRNA, Tac1-immunoreactivity and a higher magnification of respective Tac1 immunoreactivities observed in the middle panels, respectively. Bar graphs in the bottom panel **(I)** represent the number of cells expressing *tac1* counted in different brain regions of males (blue columns) and female (red columns) fish. In the anterior part of the parvocellular preoptic nucleus (PPa), there were male-dominant *tac1* mRNA expression **(A** and **B)** and Tac1 immunoreactivity **(A’** and **B’)**. In the posterior part of the parvocellular preoptic nucleus (PPp) and suprachiasmatic nucleus (SC), *tac1* mRNA expression was significantly higher in males **(C** and **D)**, while no apparent sexual dimorphism was observed in Tac1 immunoreactivity **(C’** and **D’)**. In the ventral region of the periventricular hypothalamus (Hv), numbers of cells expressing *tac1* mRNA were higher in females as compared to males (**E, F**, and **I**, P<0.01), while there was no apparent sexual morphism in Tac1 immunoreactivity **(E’** and **F’)**. In the superior raphe, more number of cells expressing *tac1* mRNA were observed in males **(G** and **G’)** as compared to females **(H** and **H’)**, but there was no significant difference (ns, **I)**. Scale bars: **A−H** and **A’−H’**: 50 µm; **A”−H”**: 20 µm. **P<0.01. See Glossary for abbreviation.

**Figure 5:**
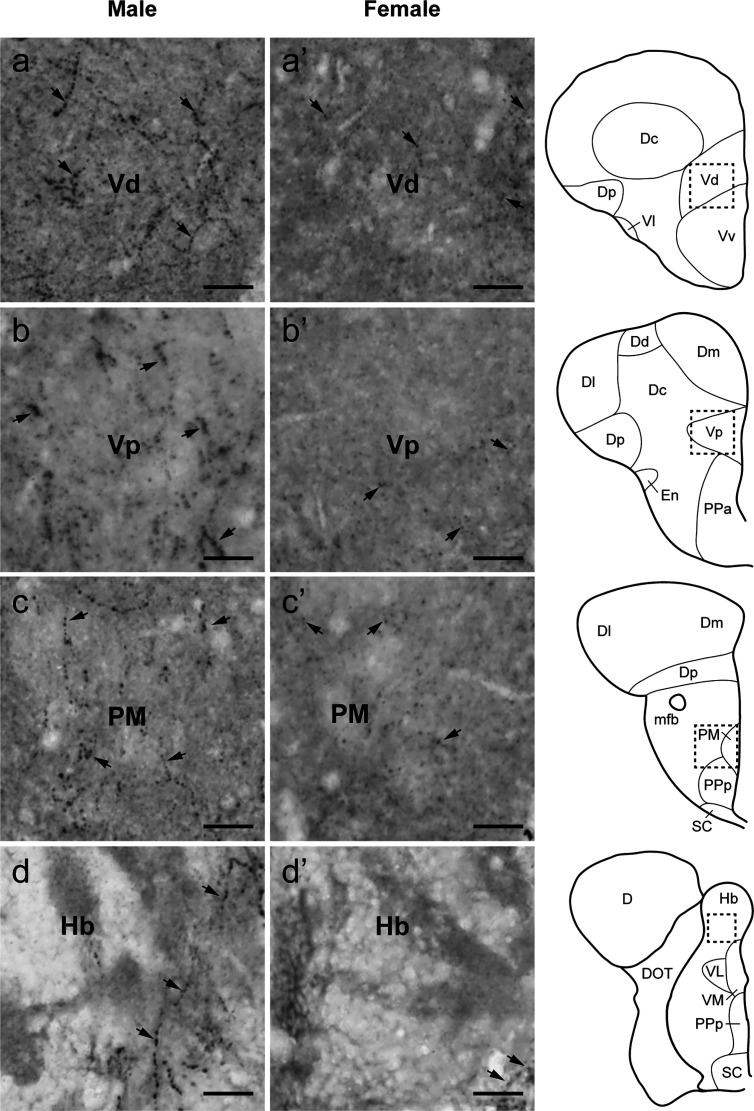
Sexual dimorphisms in Tac1-immunoreactive processes in the telencephalic and diencephalic regions. Left **(A−D)** and middle **(A’−D’)** columns represent photomicrographs of Tac1-immunoreactive processes (arrows) in the brain of males and females, respectively. Boxes with a dotted line in the schematic drawing of coronal view in the right column indicate the localization of the boundaries of the respective immunohistochemical illustrations. In the ventral part of the ventral telencephalon, more Tac1-immunoreactive processes are seen in male brains **(A−C)** as compared to females **(A’−C’)**. Similarly, more Tac1-immunoreactive processes are observed in the habenula nuclei in males as compared to female **(D**, **D’)**. Scale bars: 20 µm. See Glossary for abbreviation.

**Figure 6:**
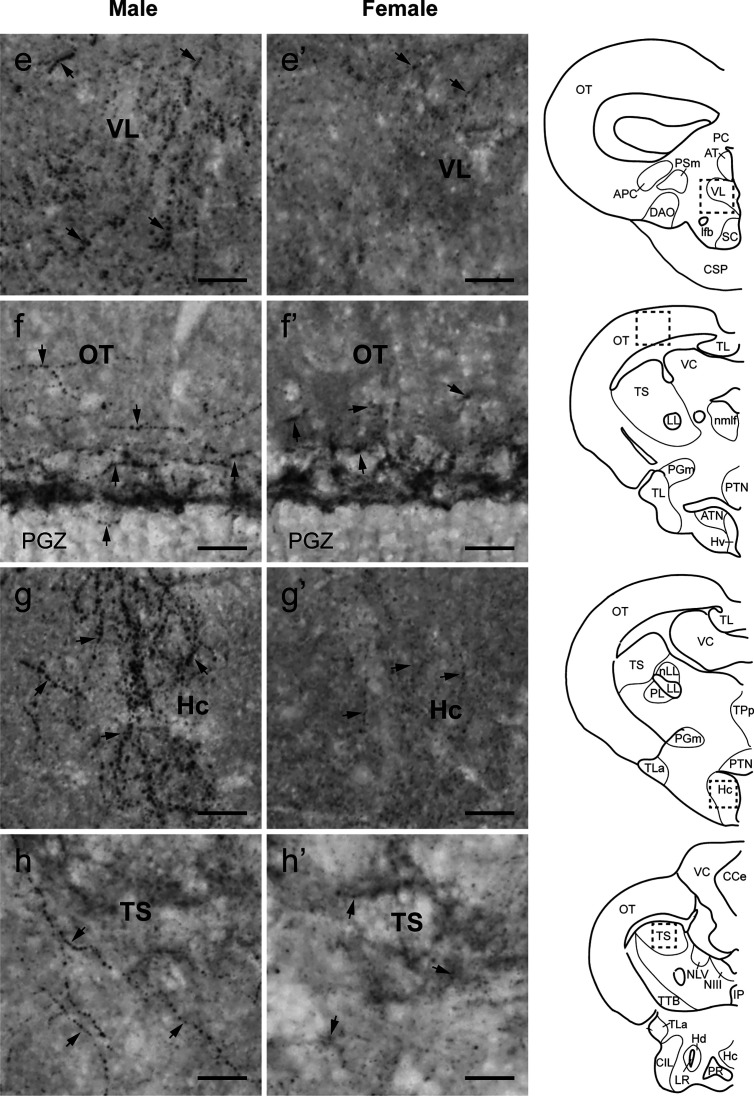
Comparison of Tac1-immunoreactive processes in the diencephalic and mesencephalic regions. Left **(E−H)** and middle **(E’−H’)** columns represent photomicrographs of Tac1-immunoreactive processes (arrows) in the brain of males and females, respectively. Boxes with a dotted line in the schematic drawing of coronal view in the right column indicate the localization of the boundaries of the respective immunohistochemical illustrations. In some diencephalic nuclei, more Tac1-immunoreactive processes are seen in male brains **(E**, **G)** as compared to females **(E’**, **G’)**. In the mesencephalic nucleus, densities of Tac1-immunoreactive processes were slightly higher in males **(F**, **H)** as compared to females **(F’**, **H’)**. Scale bars: 20 µm. See Glossary for abbreviation.

In both males and females, the similar distribution pattern of *tac1* mRNA expression was seen in the olfactory bulb, medial and posterior zones of the dorsal telencephalic area, dorsal habenula, optic tectum, ventral, dorsal and caudal zones of the periventricular hypothalamus, lateral hypothalamic nucleus, periventricular nucleus of the posterior tuberculum, nucleus of the medial longitudinal fascicle, oculomotor nerve nucleus, torus semicircularis, and interpeduncular nucleus (**Figures 1****–****3**). On the other hand, in some ventral diencephalic nuclei such as the anterior and posterior parts of the parvocellular preoptic nucleus (PPa/PPp), and suprachiasmatic nucleus (SC), abundant expression of *tac1* mRNA was observed in only males but not in females (**Figures 1–A, A’–C’**, and **4A−D**). This significant difference was also confirmed by quantitative analysis, which showed the significantly higher number of *tac1* expressing cells in the PPa/PPp region (P=0.007, Cohen’s *d* = 4.201) and SC (P=0.002, Cohen’s *d*=5.665) in males as compared to females (**Figures 4C, D, I**). Similarly, male-dominant distribution of Tac1-immunoreactive cells was seen in the PPa (**Figures 4A’**, **B’**) but not in the PPp (**Figures 4C’**, **D’**). We also found male-dominant expression of *tac1* mRNA and Tac1-immunoreactivities in the superior raphe (**Figures 3J, J’** and **4G−H”**). However, the difference in the superior raphe was not statistically significant (P=0.144, Cohen’s *d*=1.479; **Figure 4I**). We also noted that the number of *tac1* expressing cells in the ventral zone of the periventricular hypothalamus was significantly higher in females as compared to males (P=0.001, Cohen’s *d*=6.368; **Figures 1D, D’** and **4E, F, I**). In addition, higher densities of Tac1-immunoreactive processes were also seen in the telencephalon, diencephalon, and in the hindbrain regions (**Figures 5**, **6**). To enhance the immunoreactivity of Tac1 in female brains, Tac1-immunoreactivity were examined in the brain of female fish treated with colchicine. However, there was no difference in Tac1 immunoreactivities between colchicine- and water-treated brain (data not shown). Pre-absorption of the primary antibody with antigen peptides mostly eliminated immunoreactivity (**Supplementary Figures 1A, B**). Furthermore, the dot-blot assay for the Tac1 antibody showed immunoreactivity against Tac1 but not against SP (**Supplementary Figure 1C**), confirming the specificity of the Tac1 antibody to Tac1.

### Association Between Tac1 Processes and GnRH3 or *kiss2* Cells

**Figure 7:**
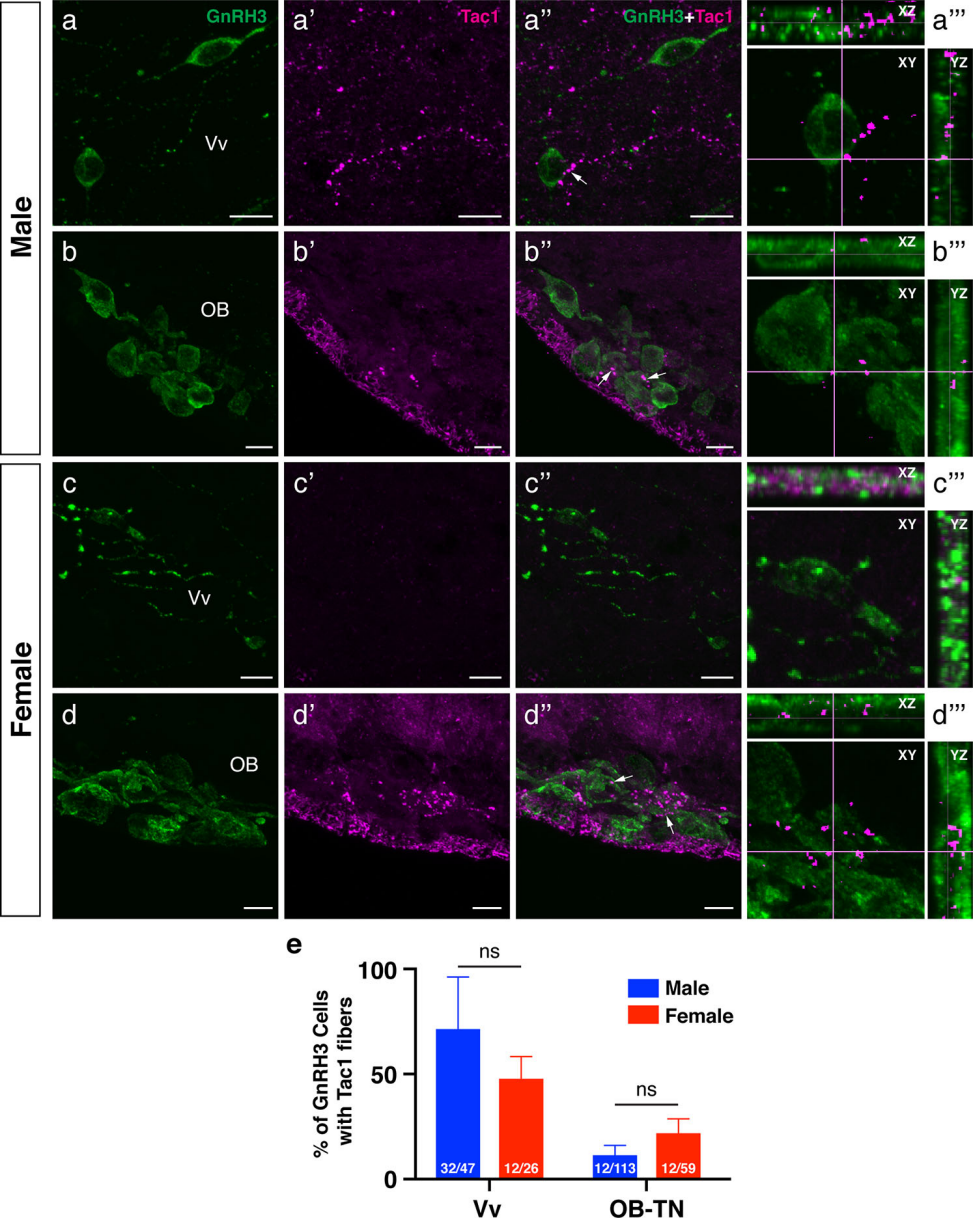
Neuronal association between Tac1-immunoreactive processes and GnRH3 cell soma in the male and female zebrafish. Photomicrographs for immunofluorescence of GnRH3 (1^st^ column, **A−D**), and Tac1 (2^nd^ column, **A’−D’**) were merged (3^rd^ column, **A”−D”**) to elucidate the possible neuronal association (arrows) between Tac1 and GnRH3 neurons. A representative GnRH3 cell soma accompanied by Tac1 processes was further examined by two-dimensional confocal scanning in XY, XZ, and YZ plane (4^th^ column, **A’”−D’”**). Bar graphs in the bottom panel **(E)** show the percentage of GnRH3 cells accompanied by Tac1 processes in males (blue columns) and females (red columns) in the ventral nucleus of ventral telencephalic area (Vv) and olfactory-terminal nerve (OB-TN) region. Numbers in each columns indicate the number of GnRH3 cells accompanied by Tac1 processes and total GnRH3 numbers counted. In the Vv, more Tac1-immunoreactive processes (*magenta*) are in proximities of GnRH3 cells (*green*) in males **(A−A’”)** as compared to females **(C−C’”)**, but there was no significant difference in the percentage of GnRH3 cells between males and females **(E)**. In the olfactory bulb (OB), OB-GnRH3 neurons are accompanied by Tac1 processes in both males **(B−B’”)** and females **(D−D’”)**, and there was no significant sex difference in the percentage of GnRH3 cells **(E)**. ns, non-significant. Scale bars: 10 µm.

**Figure 8:**
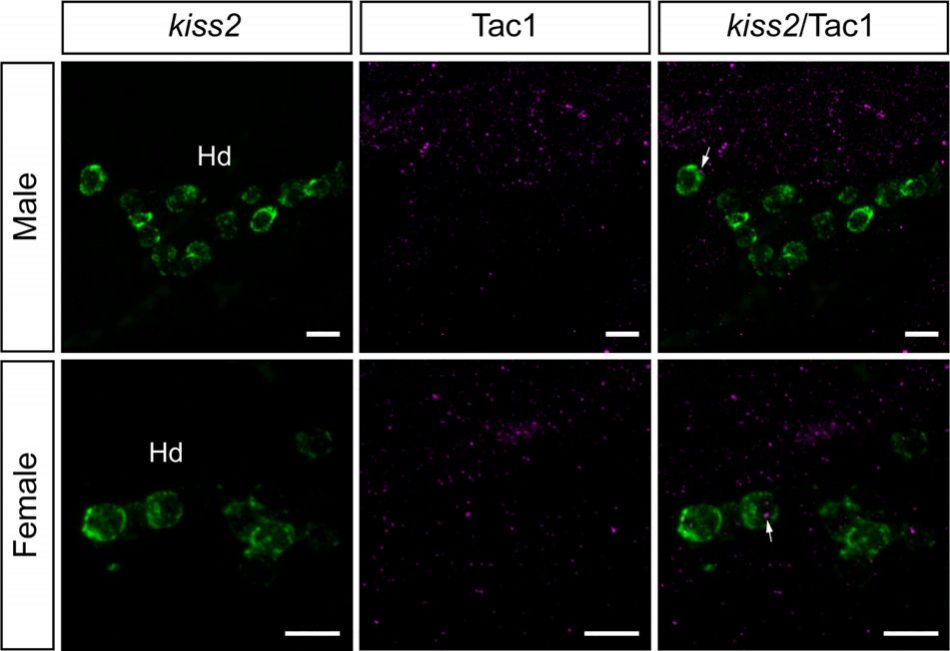
Neuronal association between Tac1-immunoreactive processes and *kiss2* cell soma in the male and female zebrafish. Photomicrographs for mRNA expression of *kiss2* (1^st^ column), and Tac1-immunoreactivity (2^nd^ column) were merged (3^rd^ column) to elucidate the possible neuronal association between Tac1 and *kiss2* neurons. Occasionally, some *kiss2* mRNA containing cells (*green*) are accompanied by Tac1 processes (*magenta*, arrows) in the dorsal zones of periventricular hypothalamus (Hd). However, they do not seem to form close associations based on the results of confocal scanning (data not shown). Scale bars: 10µm.

To examine the potential association between Tac1-immunoreactive processes and GnRH3 or *kiss2* neurons, double-labeling was performed in the brain of male and female zebrafish. Tac1-immunoreactive processes were closely located to GnRH3 cells in the ventral nucleus of ventral telencephalic area and the olfactory bulb in males (**Figures 7A-A’”**, **B-B’”**) and females (**Figures 7C-C’”**, **D-D’”**). In the ventral nucleus of ventral telencephalic area, the percentage of GnRH3 cells accompanied by Tac1 processes were higher in males (32/47 cells) as compared to females (12/26 cells), but it was statistically insignificant (P=0.203, Cohen’s *d*=1.243; **Figure 7E**). In the olfactory bulb, the percentage of GnRH3 cells accompanied by Tac1 processes were slightly higher in females (12/59 cells) as compared to males (12/113 cells), but it was statistically insignificant (P=0.093, Cohen’s *d*=1.792; **Figure 7E**). In the hypothalamic regions where *kiss2* neurons are present, occasionally, there were Tac1-immunoreactive processes that were seen in proximity to *kiss2* cells in males and females (**Figure 8**), but the densities of Tac1 processes were very subtle and they do not seem to form close associations based on the results of confocal scanning (data not shown).

### Expression of NK1 Receptor mRNA in the Brain Regions Containing GnRH3 and *kiss2* Neurons

**Figure 9:**
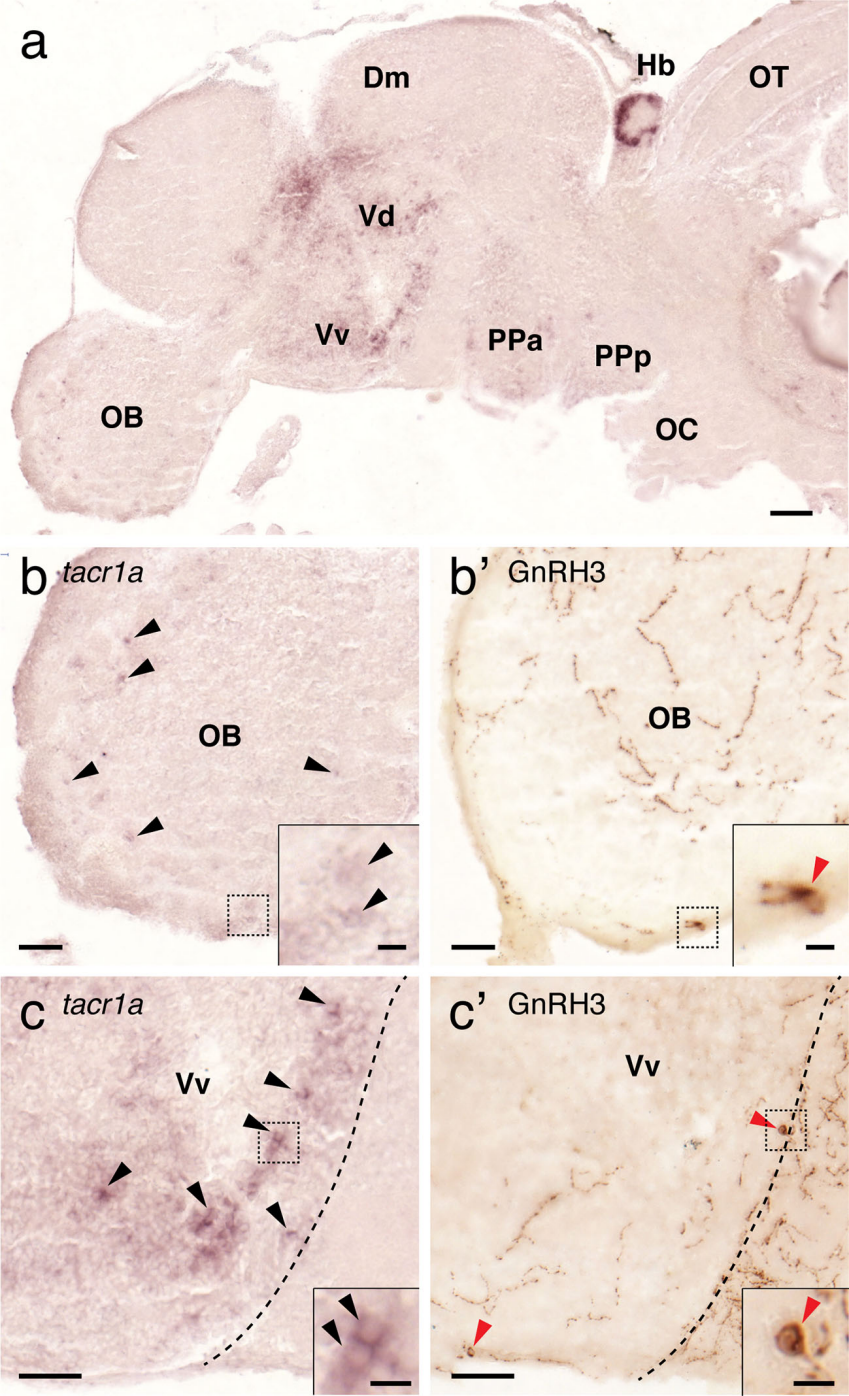
Expression of *tacr1a* mRNA in the forebrain regions containing GnRH3 cells. In the forebrain, *tacr1a* mRNA is expressed in the olfactory bulb (OB), dorsal (Vd) and ventral (Vd) regions of the ventral telencephalon, and anterior part of the parvocellular preoptic nucleus (PPa) **(A)**. In the OB, weak expression of *tacr1a* mRNA was seen in the glomerular layer (b, *black* arrowheads), and in the primary olfactory fiber layer of the olfactory bulb where GnRH3 neurons are present (**B**’, *red* arrowheads). In the Vv, where another GnRH3 cell population is present (**C’**, *red* arrowheads), *tacr1a* mRNA is broadly expressed (**C**, *black* arrowheads). Doted box indicates the location of the inset. Scale bars: **(A)**, 100 µm; **(B, B’, C, C’)**, 50 µm; insets, 10 µm.

To elucidate possible direct action of Tac1 on GnRH3 and Kiss2 neurons, expression of NK1 receptor (*tacr1a*) mRNA was examined in the brain regions where GnRH3 or *kiss2* cells are present. *In situ* hybridization showed expression of *tacr1a* mRNA in the forebrain regions including the olfactory bulb, ventral telencephalic area and the parvocellular preoptic nucleus (**Figure 9A**). In the olfactory bulb, a few *tacr1a* expressing cells were seen along the border between the glomerular layer and the external cellular layer of the olfactory bulb (**Figure 9B**). *tacr1a* mRNA was also weakly expressed in the primary olfactory fiber layer, where olfactory-terminal nerve GnRH3 cells are present (**Figures 9B**, **B’**). In the ventral nucleus of the ventral telencephalic area, where preoptic-GnRH3 neurons are present, *tacr1a* mRNA was broadly expressed (**Figures 9C, C’**).

**Figure 10:**
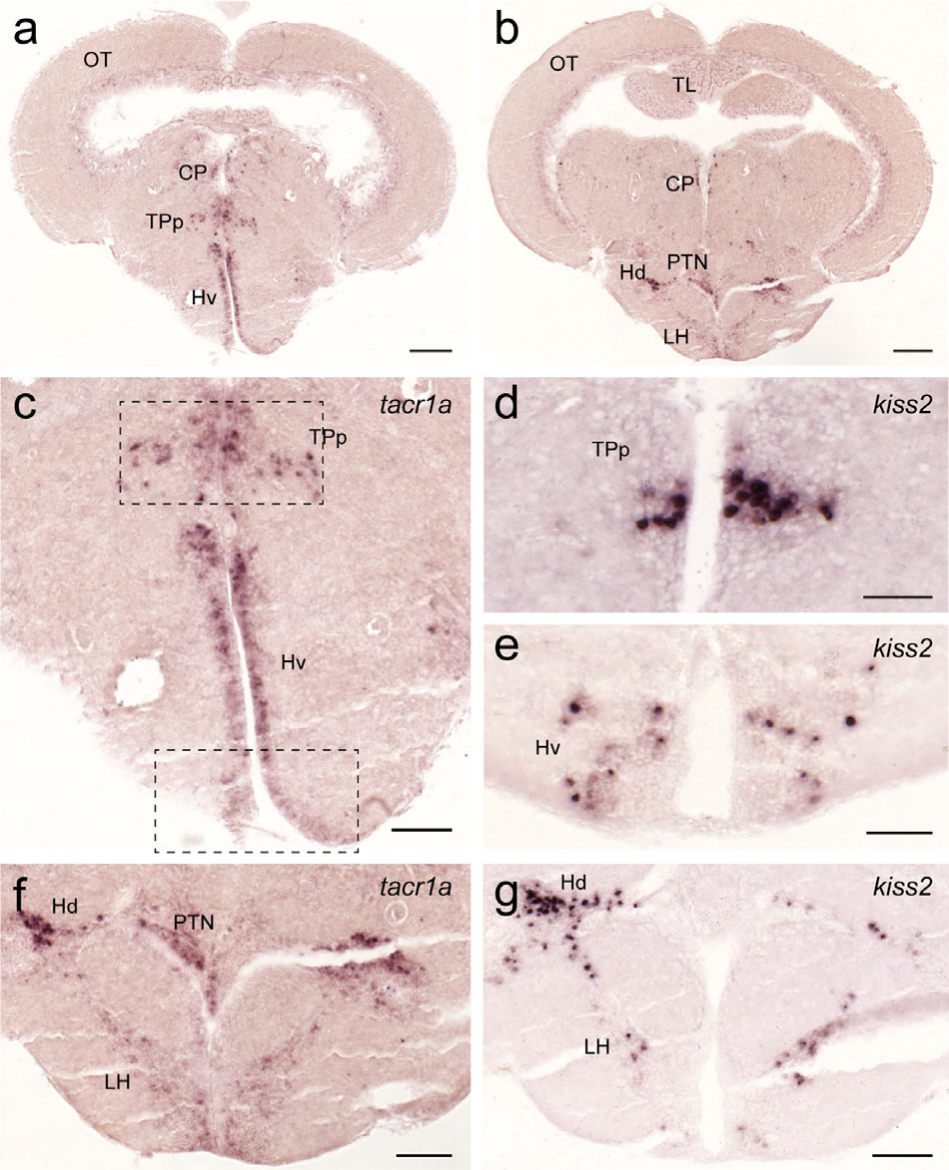
Expression of *tacr1a* mRNA in the hypothalamic regions containing *kiss2* cells. In the hypothalamic regions, *tacr1a* mRNA is expressed in several regions **(A**, **C)** including the ventral nucleus of the ventral telencephalic area and the periventricular nucleus of the posterior tuberculum (dotted box), where *kiss2* neurons are present **(D**, **E)**. In addition, *tacr1a* expression was also seen in the lateral hypothalamic nucleus towards the dorsal zone of the periventricular hypothalamus **(B**, **F)**, where another group of *kiss2* neurons is present **(G)**. Scale bars: **(A**, **B)**, 200 µm; **(C, F, G)**, 100 µm; **(D**, **E)**, 50 µm. See Glossary for abbreviation.

In the hypothalamus, *tacr1a* expression was seen along the diencephalic ventricle including the ventral nucleus of the ventral telencephalic area, posterior tuberal nucleus, central posterior thalamic nucleus (**Figure 10A**), and in the periventricular nucleus of the posterior tuberculum where *kiss2* neurons are located (**Figures 10C–E**). In addition, *tacr1a* expression was also seen in the lateral hypothalamic nucleus towards the dorsal zone of the periventricular hypothalamus, where another group of *kiss2* neurons is present (**Figures 10B, F, G**).

## Discussion

An apparent male-dominant sexually dimorphic expression of *tac1* mRNA and Tac1-immunoreactivities was observed in the ventral telencephalic area, similar to mammals and other fish species (32, 34). In mammals, the male-dominant expression pattern of SP has also been reported in the medial amygdala and basal ganglia regions (30, 31). Although these brain structures are morphologically indistinguishable in the fish species, several hodological characterizations based on neuroanatomy, molecular and neurochemical markers suggest that the ventral subpallium, the ventral part of the ventral telencephalic area of the non-mammalian brain, may represent the basal ganglia (69–71). On the other hand, the teleostean medial zone of the dorsal telencephalon is considered to contain the homologue of the pallial amygdala because of their involvement in spatial cognition and fear expression in fish (72, 73). Recently, based on morphological and functional assays in zebrafish, the intermediate ventral telencephalic nucleus has been proposed as the teleostean medial amygdala (74). Since SP has been known as a common marker of the basal ganglia and amygdala (69, 75), male-dominant SP expression in the ventral part of the ventral telencephalic regions could be functional evidence supporting the ventral subpallium as the teleostean basal ganglia (and amygdala). In the zebrafish, we did not find any apparent sex differences in the diencephalon and mesencephalon, except for a female dominant *tac1* expression in the ventral zone of periventricular hypothalamus. In humans, the number of SP cells in the hypothalamic infundibular (or arcuate) nucleus is higher in postmenopausal woman compared with age-matched men (49). This suggests that SP/NKA population in the basal forebrain is male dominant, while the hypothalamic SP/NKA population is female dominant. In the rhombencephalon, there was a stronger expression of *tac1* mRNA in the superior raphe in males as compared to females, although the difference was insignificant. In human, SP is expressed in serotonergic neurons in the raphe nucleus (76, 77), and serotonin-immunoreactive signal intensity in the rat dorsal raphe is male-dominant (78). Therefore, sexually dimorphic *tac1* expressing neurons in the raphe could be serotonergic.

Sexually dimorphic expression pattern of Tac1 cells and processes indicate that gonadal steroids may exert either organizational or activational effects on the Tac1 system. In an electric fish, *Apteronotus leptorhynchus*, treatment with androgens increased SP-immunoreactive cell numbers in the ventral telencephalon and hypothalamic regions (79, 80). Similarly, in rodents, administration of anabolic androgenic steroids increases SP immunoreactivity in several brain regions including the amygdala and hypothalamus (81–83). Alternatively, it is also possible that the lesser expression of *tac1* in females could be due to the inhibitory action of estrogens on SP neurons. In primates and humans, there is a negative correlation between plasma levels of estradiol and SP during the follicular phase (84). Similarly, in female rats, the concentration of SP in the bed nucleus and ventromedial nucleus of the hypothalamus is decreased during the estrus stage as compared to diestrus and proestrus stages (28). On the other hand, in some mammalian species, estrogen upregulates hypothalamic SP (32, 85–89). In castrated rats, estrogen increases *Tac1* mRNA levels in the hypothalamus, while androgen only upregulates *Tac1* mRNA levels in the anterior pituitary but it does not affect hypothalamic *Tac1* mRNA levels (32). In female medaka, higher aromatase expression and activity are seen in the periventricular hypothalamus as compared to males (90). This suggests that the higher expression of *tac1* in the periventricular hypothalamus of female zebrafish could be under estrogenic regulation. In the brain of zebrafish, estrogen and androgen receptors are highly expressed in the ventral telencephalic regions where male dominant Tac1 neurons were found (91–93). In medaka, three types of estrogen receptors (*esr1*, *esr2a*, and *esr2b*) and two types of androgen receptors (*ara* and *arb*) genes exhibited brain region-specific sexually dimorphic expression patterns (94). In particular, in the ventral telencephalic-preoptic regions, *esr2a* exhibits male-dominant, while *esr1*, *esr2b*, and *arb* exhibit female-dominant expression (94). However, in zebrafish, there is no apparent sexual dimorphism in androgen receptor expression in the ventral telencephalic regions (93). Although the exact reason for this discrepancy remains unknown, male-dominant expression of Tac1 in the ventral telencephalic region could be a result of the organizational effect of sex steroids during brain development. These results suggest that Tac1 expression could be regulated by steroids in brain regions- and reproductive conditions-dependent manners.

In the present study, we generated an antibody against the carboxyl-terminal region of the zebrafish Tac1, which has low homology with other tachykinin family of peptides. Tac1-immunoreactivities in neurons and processes were widely observed in the brain, which was successfully eliminated by pre-absorption with the antigen peptide. Since our antibody is generated against the carboxyl-terminal region encoded by exon-5 of *tac1* gene, which exists in all splicing variants, this antibody should recognize all precursors produced by different splice variant mRNAs of *tac1* gene. Furthermore, Tac1-immunoreactive cells can only be found in brain regions where *tac1* mRNA-expressing cells are present suggesting that cross-reactivity of the Tac1 antibody to other tachykinins is likely to be low. In some brain regions, Tac1-immunoreactivity appeared to be considerably lower than the densities of *tac1* expression. We also observed Tac1-immunoreactivities in fish administered with colchicine (to enhance the staining), but we did not find any apparent difference in the intensity of Tac1-immunoreactivity (data not shown). Although the exact reason for this discrepancy remains unclear, it may suggest the presence of differential translational activity of *tac1* mRNA among Tac1 cell populations. In fact, biosynthesis of Tac1 peptides has been suggested to be modulated by region specific translation or processing mechanism in the rat brain (95). Therefore, Tac1 peptide content levels could vary depending on the animal’s physiological condition or brain regions.

Using the zebrafish Tac1 antibody, we also found that GnRH3 neurons are closely accompanied by Tac1-immunoreactive processes in the forebrain. Further, we also localized NK1 receptor (*tacr1a*) mRNA in the forebrain regions containing GnRH3 cells, suggesting possible expression of NK1 receptor in GnRH3 cells. On the other hand, although *tacr1a* expression was seen in most of the hypothalamic regions containing *kiss2* cells, Tac1-immunoreactive processes did not closely accompany *kiss2* cells. These results imply that Tac1 peptides could act directly on GnRH3 cells but not on Kiss2 cells. However, whether *tacr1a* is expressed in GnRH3 and Kiss2 cells, and Tac1 processes form synaptic action on GnRH3/Kiss2 cells remain to be investigated.

In the ventral nucleus of ventral telencephalic area, more GnRH3 neurons are accompanied by Tac1 processes in males as compared to females. In some fish, sex-dependent expression pattern of GnRH has been reported. In wrasses, there are intra- and intersexual dimorphisms in the number of GnRH neurons in the preoptic area (96). In medaka, GnRH1 and GnRH3 genes exhibit brain region-specific sexually-dimorphic expression patterns (97). As preoptic-GnRH3 is considered to act as the hypophysiotropic form in the zebrafish (44, 98, 99), Tac1 may be responsible for a male-typical pattern of regulation of GnRH3 and GnRH3-dependent LH secretion in fish. In addition, GnRH3 cells in the olfactory bulbar-terminal nerve (OB-TN) region are also accompanied by Tac1 processes with a slight female dominance. In teleosts, OB-TN GnRH3 neural population has been shown to regulate social behaviors such as aggression, sexual behavior and partner preference (100–102). In mammals, NK1 receptor-mediated SP neurotransmission has been implicated in the control of social behaviors such as aggression, sexual behavior and anxiety (103–105). Hence, Tac1 signaling may be involved in OB-TN GnRH3 dependent social functions.

In summary, we found clear male-dominant sexually dimorphic expression pattern of *tac1* in the ventral telencephalon and preoptic regions of zebrafish. Further, we also found that Tac1 processes are in proximities of GnRH3 cells but not with *kiss2* cells. Therefore, in the zebrafish, sexually dimorphic Tac1 neuronal populations might directly act on GnRH3 neurons, independent of their influence on kisspeptin. However, it remains unknown whether there is any sex difference in the modulatory effect of SP/NKA on GnRH system. Although there was no apparent sex difference in number of GnRH3 cells accompanied by Tac1 processes, in males preoptic-GnRH3 neurons are in proximity with more Tac1 processes, while in females, more GnRH3 neurons are accompanied by Tac1 processes in the olfactory bulb. Hence, it can be hypothesized that SP/NKA may have a gender- and brain region-biased influence on GnRH3 neurons in the zebrafish.

## Data Availability Statement

The raw data supporting the conclusions of this article will be made available by the authors, without undue reservation, to any qualified researcher.

## Ethics Statement

The animal study was reviewed and approved by Animal Ethics Committee of Monash University (Approval number: MARP/2012/120).

## Author Contributions

SO and ISP designed research. SO and ISP created antisera for zebrafish TAC1. PNR and RA performed the experiments. PNR and SO analyzed the data, and PNR, SO, and ISP wrote the paper. All authors contributed to the article and approved the submitted version.

## Funding

This work is supported by Monash University Malaysia (M-NEU-RS-014); Malaysian Ministry of Higher Education, FRGS/2/2010/ST/MUSM/03/02 (to SO); and FRGS/1/2013/SKK01/MUSM/03/02 (to ISP) and Malaysian Ministry of Science and Technology and Innovation, 02-02-10-SF0161 (to ISP).

## Conflict of Interest

The authors declare that the research was conducted in the absence of any commercial or financial relationships that could be construed as a potential conflict of interest.

## Supplementary Material

Supplementary Figure 1 Comparison of Tac1 immunoreactivity with and without pre-absorption with the antigen peptide. **(A)** Photomicrographs of Tac1-immunoreactive fibers (arrows) in the dorsal region of the ventral telencephalon (Vd), of which immunoreactivity was mostly diminished by the pre-absorption with the antigen peptide **(B)** Scale bars: 50 µm. **(C)** Dot-blot analysis showed that anti-SP antibody reacted to human SP, but not to the zebrafish Tac1 antigen peptide (first column), while the anti-zebrafish Tac1 antibody strongly reacted to the Tac1 antigen peptide, but not to human SP peptide (second column). Pre-absorption with the antigen peptide diminished the immunoreactivity to the Tac1 (third column).

## Glossary

**Table:**

Abbreviations	Name of brain area
AON	anterior octaval nucleus
APC	accessory pretectal nucleus
AT	anterior thalamic nucleus
ATN	anterior tuberal nucleus
Cans	ansulate commissure
CC	cerebellar crest
CCe	cerebellar corpus
GC	central gray
CIL	central nucleus of inferior lobe
CM	mammillary body
CP	central posterior nucleus of dorsal thalamus
CSP	commissura postoptica
D	dorsal telencephalic area
DAO	dorsal accessory optic nucleus
Dc	central zone of dorsal telencephalic area
Dd	dorsal zone of dorsal telencephalic area
dHb	dorsal habenula
DIL	diffuse nucleus of inferior lobe
Dl	lateral zone of dorsal telencephalic area
Dm	medial zone of dorsal telencephalic area
DOT	dorsomedial optic tract
Dp	posterior zone of dorsal telencephalic area
DTN	dorsal tegmental nucleus
DV	descending trigeminal root
EG	granular eminence
En	entopeduncular nucleus
FR	fasciculus retroflexus
Hb	habenula
Hc	caudal periventricular hypothalamic zone
Hd	dorsal periventricular hypothalamic zone
Hv	ventral periventricular hypothalamic zone
IP	interpeduncular nucleus
LC	locus coeruleus
lfb	lateral forebrain bundle
LH	lateral hypothalamic nucleus
LL	lateral lemniscus
LLF	lateral longitudinal fascicle
LR	lateral recess of diencephalic ventricle
MB	midbrain
mfb	medial forebrain bundle
mlf	medial longitudinal fascicle
NIII	oculomotor nucleus
NLL	nucleus of the lateral lemniscus
NLV	nucleus lateralis valvulae
nmlf	nucleus of the medial longitudinal fascicle
NVmd	dorsal part of trigeminal motor nucleus
NVmv	ventral part of trigeminal motor nucleus
OC	optic chiasm
OT	optic tectum
PC	posterior commissure
PGm	medial preglomerular nucleus
PGZ	periventricular gray layer of the optic tectum
PL	perilemniscal nucleus
PM	magnocellular preoptic nucleus
PPa	anterior part of parvocellular preoptic nucleus
PPp	posterior part of parvocellular preoptic nucleus
PR	posterior recess of the diencephalic ventricle
PSm	magnocellular part of superficial pretectum
PSp	parvocellular part of superficial pretectum
PTN	posterior tuberal nucleus
RT	rostral tegmental nucleus
SC	suprachiasmatic nucleus
SGT	secondary gustatory tract
SR	superior raphe nucleus
SRF	superior reticular formation
TL	torus longitudinalis
TLa	nucleus of the lateral torus
TPp	periventricular nucleus of posterior tuberculum
TS	torus semicircularis
TTB	tecto-bulbar tract
TTBc	tractus tectobulbaris cruciatus
VC	valvula cerebelli
Vd	dorsal nucleus of ventral telencephalic area
vHb	ventral habenula
VL	ventrolateral thalamic nucleus
Vl	lateral nucleus of ventral telencephalic area
VM	ventromedial thalamic nucleus
Vmv	ventral motor root of the trigeminal nerve
Vp	postcommissural nucleus of ventral telencephalic area
Vv	ventral nucleus of ventral telencephalic area
