# Supplementary figures and images for: Sexual Dimorphic Distribution of Hypothalamic Tachykinin1 Cells and Their Innervations to GnRH Neurons in the Zebrafish
Source: Front Endocrinol (Lausanne). 2021 Mar 3;11:534343. doi: 10.3389/fendo.2020.534343 (PMC7982876; doi:10.3389/fendo.2020.534343)

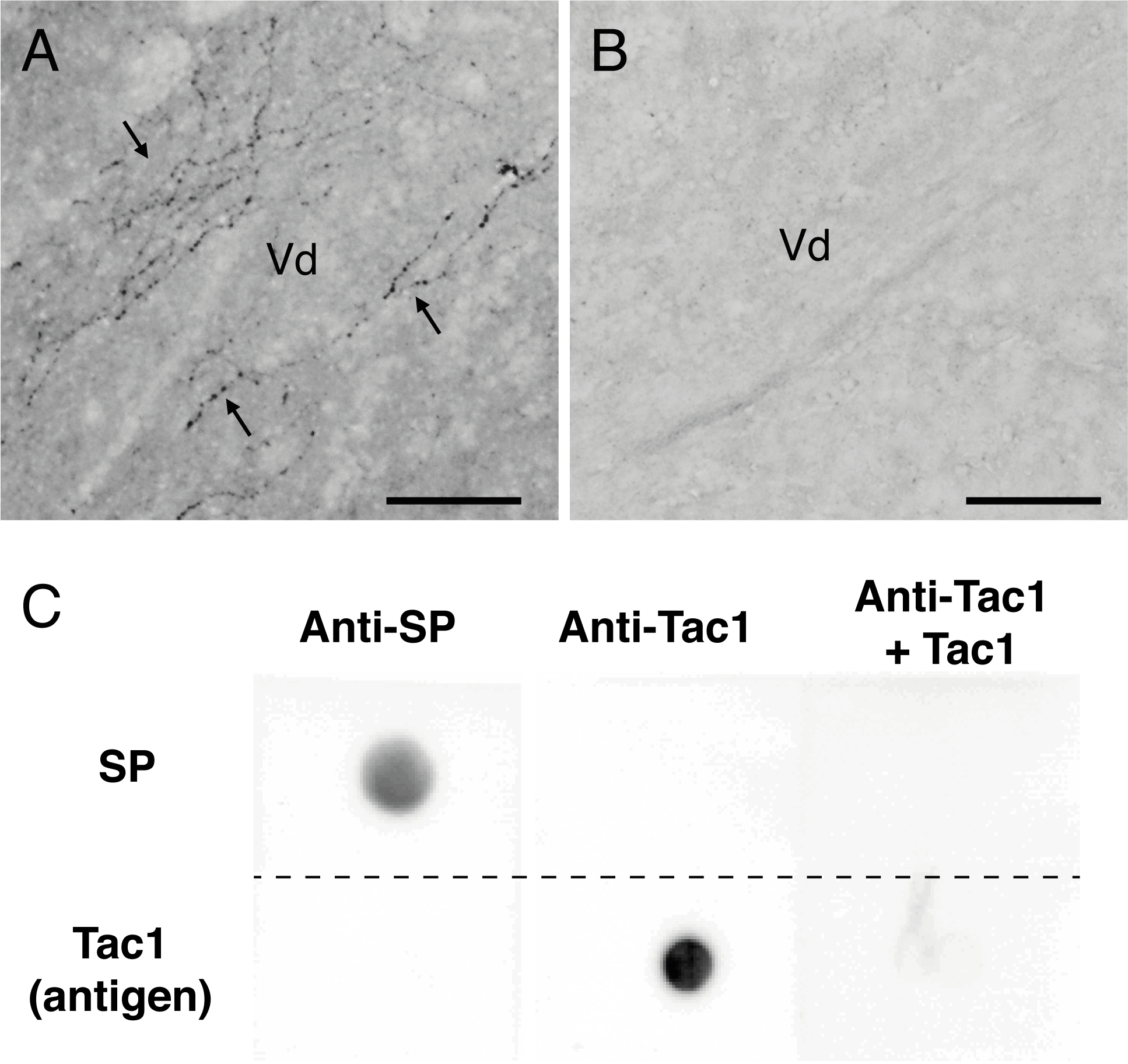

Supplement: Supplementary Figure 1 — Comparison of Tac1 immunoreactivity with and without pre-absorption with the antigen peptide. (A) Photomicrographs of Tac1-immunoreactive fibers (arrows) in the dorsal region of the ventral telencephalon (Vd), of which immunoreactivity was mostly diminished by the pre-absorption with the antigen peptide (B) Scale bars: 50 µm. (C) Dot-blot analysis showed that anti-SP antibody reacted to human SP, but not to the zebrafish Tac1 antigen peptide (first column), while the anti-zebrafish Tac1 antibody strongly reacted to the Tac1 antigen peptide, but not to human SP peptide (second column). Pre-absorption with the antigen peptide diminished the immunoreactivity to the Tac1 (third column). [file Image_1.tif]
